# Involvement of *Met* and *Kr-h1* in JH-Mediated Reproduction of Female *Bactrocera dorsalis* (Hendel)

**DOI:** 10.3389/fphys.2018.00482

**Published:** 2018-05-04

**Authors:** Yong Yue, Rui-Lin Yang, Wei-Ping Wang, Qi-Hao Zhou, Er-Hu Chen, Guo-Rui Yuan, Jin-Jun Wang, Wei Dou

**Affiliations:** ^1^Key Laboratory of Entomology and Pest Control Engineering, College of Plant Protection, Southwest University, Chongqing, China; ^2^Academy of Agricultural Sciences, Southwest University, Chongqing, China

**Keywords:** juvenile hormone, reproduction, *Kr-h1*, methoprene, *Bactrocera dorsalis*

## Abstract

Juvenile hormone (JH) prevents metamorphosis during insect larval stages and promotes adult reproductive processes. Krüppel-homolog 1 (Kr-h1), a zinc finger transcription factor assumed to be induced by JH via the JH receptor methoprene-tolerant (Met), mediates the antimetamorphic effect of JH in insects, but its function in JH-mediated reproductive processes has not been fully explored. In this study, *Met* and *Kr-h1* involved in the JH signaling pathway were first cloned and identified from the oriental fruit fly, *Bactrocera dorsalis*, an important pest infesting fruit and vegetables worldwide. Subsequent spatiotemporal expression analysis revealed that *Met* and *Kr-h1* were both highly expressed in 7-day-old adults and fat body of female adults, respectively. Treatment with a JH analog (methoprene) significantly induced the expression of JH signaling and *vitellogenin* (*Vg*) genes and accelerated ovary development. RNA interference (RNAi) further revealed that either *Met* or *Kr-h1* depletion at the adult stage of *B. dorsalis* impeded ovary development, with significantly lower egg production noted as well. In addition, rescue through methoprene application after RNAi stimulated the expression of JH signaling and *Vg* genes. Although there were still differences in ovary phenotype between rescued insects and the pre-RNAi control, ovary redevelopment with a larger surface area was observed, consistent with the spatiotemporal expression and phenotypes recorded in the original methoprene experiment. Our data reveal the involvement of Met and *Kr-h1* in insect vitellogenesis and egg production, thus indicating the crucial role of the JH signaling pathway in insect reproduction.

## Introduction

Juvenile hormone (JH) is one of the most important hormones regulating insect development, metamorphosis, and reproduction (Riddiford et al., 2010; Hiruma and Kaneko, 2013; Song et al., 2014). This hormone, which is present during larval molting to prevent immature insects from transforming into adults, reappears in adults to regulate female reproductive maturation. To date, the JH signaling pathway has been a prominent topic in insect research. However, recent efforts have largely been limited to the identification of JH receptors. Methoprene-tolerant (Met), a receptor of JH, has recently been systematically characterized (Jindra et al., 2013); in contrast, the molecular mechanism underlying the JH signaling pathway is far from clear.

Methoprene-tolerant is a basic-helix-loop-helix (bHLH)-Per-Arnt-Sim (PAS) transcription factor, first reported in *Drosophila melanogaster* (Jindra et al., 2013). An *in vitro* study revealed that Met mediates transcription in a JH-dependent manner and binds to JHIII at nanomolar levels (Miura et al., 2005), suggesting that Met plays a crucial role in JH signaling in *Drosophila*. However, Met mutants are viable, suggesting that another gene affords functional redundancy. Further genetic evidence has definitively established germ cell-expressed (Gce)/Met in a JH receptor role (Jindra et al., 2015). In contrast, *Tribolium castaneum* has a single Met-like ortholog. Silencing of *Met* in early-instar larvae of *T. castaneum* by RNA interference (RNAi) leads to the production of miniature pupae or heterochronic larva–pupa intermediates (Konopova and Jindra, 2007). *Met* RNAi in the final larval instar of *T. castaneum* significantly accelerates the development of pupae and adults (Parthasarathy et al., 2008). The generated phenotype is similar to the phenotype of JH deficiency caused by CA ablation or genetic manipulation (Daimon et al., 2012).

As an early JH-response gene downstream of *Met, Krüppel-homolog 1* (*Kr-h1*) encodes a key transcription factor that regulates insect metamorphosis in the JH signaling pathway (Marchal et al., 2014). Kr-h1 has been shown to be the direct target of Met (Kayukawa et al., 2012; Shin et al., 2012). Following the identification of Met as a JH receptor, a regulatory model involving JH-Met-Kr-h1 has been gradually developed (Konopova et al., 2011; Huang et al., 2013). During *Drosophila* metamorphosis, increased expression of *Kr-h1* brought about by JH through the receptors Met and Gce maintains larval morphology and regulates larval metamorphosis and development (Abdou et al., 2011). Interruption of *Kr-h1* in penultimate-instar larvae of *T. castaneum* results in precocious metamorphosis, an effect similar to that caused by the depletion of *Met* or the gene coding the JH acid methyltransferase, which is involved in JH biosynthesis (Konopova and Jindra, 2007; Minakuchi et al., 2009). Treatment with exogenous JH or JH analogs can regulate the expression level of *Kr-hl*, thereby maintaining larval morphology or inhibiting metamorphosis (Feyereisen and Jindra, 2012).

In addition to its roles in juveniles, JH stimulates a variety of physiological functions in adult insects, including previtellogenic development, vitellogenesis, and oogenesis (Wyatt and Davey, 1996). In contrast, the physiological mechanism of the JH signaling pathway in insect reproduction is poorly understood. In several model insects, both JH and 20-hydroxyecdysone (20E) are involved in reproduction, and JH mediates vitellogenesis in *D. melanogaster* (Riddiford, 2012), previtellogenic development in *Aedes aegypti* (Raikhel et al., 2002), and vitellogenin (Vg) in *T. castaneum* (Parthasarathy et al., 2010a,b). In contrast, JH acts independently of 20E to regulate vitellogenesis and oocyte maturation in many other insect species including *Pyrrhocoris apterus* and *Locusta migratoria* as well as *Blattella germanica* (Wyatt and Davey, 1996; Smykal et al., 2014). The JH signaling genes *Met* and *Kr-h1* have been implicated in oogenesis. In a recent study of *L. migratoria, Kr-h1* RNAi significantly inhibited the development of primary oocytes and ovaries (Song et al., 2014). During JH-mediated previtellogenic development of the mosquito *A. aegypti, Kr-h1* transcription levels are regulated by the *Met* complex, but the exact physiological mechanism behind the involvement of *Kr-h1* in this process has not been revealed (Zhu et al., 2010; Shin et al., 2012). In *P. apterus*, RNAi of *Met*, but not *Kr-h1*, has been found to block ovarian development and to suppress *Vg* gene expression in the fat body (Smykal et al., 2014).

The oriental fruit fly, *Bactrocera dorsalis* (Hendel) (Diptera: Tephritidae), which is widely distributed in tropical and subtropical areas, is an extremely destructive pest of a wide variety of types of commercial fruit and vegetables worldwide, with an especially damaging impact on citrus (Clarke et al., 2005). Because of its strong propensity for invasion, *B. dorsalis* has been listed as a quarantine pest in many countries and regions. Control of this pest has mainly relied on the application of insecticides, but many populations of *B. dorsalis* have evolved high levels of resistance toward nearly all commonly used insecticide groups (Jin et al., 2011). Control of the oriental fruit fly is thus increasingly important. Insect reproduction and molting have been a focus of pest control research; consequently, clarification of the insect JH signaling pathway and related gene expression should provide new ideas that are useful for pest control. Although the JH signaling pathway has been studied systematically in model insects such as *D. melanogaster* and *T. castaneum*, relevant information is limited in oriental fruit flies.

In the current study, we first cloned and identified the full-length cDNAs of *BdMet* and *BdKr-h1*. We then analyzed the spatiotemporal expression patterns of these genes at different stages and in various tissues of *B. dorsalis* adults. Treatment with the JH analog methoprene and reverse genetics were both applied to explore the function of *BdKr-h1* in *B. dorsalis* reproduction. We found that *Kr-h1* mediates the effect of JH on the induction of vitellogenesis. Silencing of *BdMet* or *BdKr-h1* resulted in poor egg production due to drastic reduction in Vg expression as well as severely impaired oocyte maturation and ovarian growth. Our data reveal a critical role of *Kr-h1* in insect ovary development and thus provide new insights into JH signaling transduction during insect reproduction.

## Materials and Methods

### Experimental Insects

The *B. dorsalis* population used in this study was collected from Fujian Province, China, in 2010. The adults were reared in a metal cage and fed on an artificial diet consisting of honey, sugar, yeast powder, vitamin C, and water (Wang et al., 2013). Adults for the experiments were subsequently reared at 27 ± 0.5°C and 70 ± 5% relative humidity under a 14-h light/10-h dark photoperiod (Shi et al., 2017) in a temperature-controlled room at the Key Laboratory of Entomology, Southwest University, Chongqing, China. In this study, male and female adults were separated before 4 days of age. To ensure the consistency of the experimental materials, all adults were maintained under the same conditions before starting the experiments.

### RNA Extraction, Reverse Transcription, and cDNA Synthesis

Adults were collected, frozen in liquid nitrogen, and subjected to total RNA extraction using Trizol reagent (Invitrogen Life Technologies, Carlsbad, CA, United States), in accordance with the manufacturer’s protocol. The extracted RNA was immediately dissolved in RNase-free water and checked for quality, concentration, and purity on a NanoVue UV–Vis spectrophotometer (GE Healthcare Bio-Sciences, Uppsala, Sweden) at 260 and 280 nm. RNA integrity was also checked by 1–1.5% agarose gel electrophoresis at 180 V for 16 min. Four biological replicates were conducted per treatment. Genomic DNA was removed from the RNA samples using RQI DNase (Promega, Madison, WI, United States). Finally, first-strand cDNA was synthesized from total RNA using a PrimeScript RT reagent kit (Takara, Dalian, China), following the manufacturer’s protocol.

### Molecular Cloning

Gene-specific primers for amplification of gene full-length coding regions were designed (**Supplementary Table S1**) based on published *B. dorsalis* transcriptome data (Shen et al., 2013) using Primer Premier 5.0 (Premier Biosoft International, Palo Alto, CA, United States), with DNAMAN v.6.03 (Lynnon Biosoft, San Ramon, CA, United States) being used for sequence alignment. The open-reading frame (ORF) sequences of *BdKr-h1* and *BdMet* were PCR-amplified using PrimeSTAR high-fidelity DNA polymerase (Takara, Dalian, China), in accordance with the following protocol: initial denaturation at 98°C for 2 min; followed by 35 cycles of 98°C for 15 s, 60°C for 15 s, and 72°C for 3 min; with final extension at 72°C for 10 min followed by holding at 12°C. The resulting PCR products were separated by electrophoresis on agarose gels (1.0–1.5%). PCR products of the expected size were excised from the gels, ligated into a pGEM-T Easy vector (Promega, Beijing, China), and transformed into Trans5α chemically competent cells (TransGen Biotech Co., Ltd., Beijing, China). Transformants were selected on Luria–Bertani agar plates containing 0.1% ampicillin and sequenced (BGI, Beijing, China).

### Phylogenetic Tree Construction and Sequence Analysis

To infer the evolutionary history of *B. dorsalis Kr-h1* and *Met*, Kr-h1 and Met protein sequences from various species were downloaded from the National Center for Biotechnology Information web server^1^ and aligned with sequences of BdKr-h1 and BdMet in ClustalX 2 software (Larkin et al., 2007) and JalView 2.9. A phylogenetic tree of these sequences was constructed by neighbor joining in 5.0 (Tamura et al., 2011) with 1,000 bootstrap replicates. In addition, the online software tool ProtParam^2^ was used to predict the molecular mass and isoelectric point of BdMet and BdKr-h1. Finally, the SignalP server^3^ was used to predict signal peptides.

### Quantitative Real-Time PCR (qRT-PCR)

Following first-strand cDNA synthesis of *BdKr-h1* and *BdMet* with a PrimeScript RT reagent kit (Takara, Dalian, China), qRT-PCR primers were designed (**Supplementary Table S1**) using Primer3.0 and DNAMAN v.6.03. The amplification efficiency of each pair of primers was first validated by constructing a standard curve based on a fivefold cDNA dilution series, with the data then analyzed in Biogazelle qBase. Next, the Novostar-SYBR Supermix (Novoprotein, Shanghai, China) was used for qRT-PCR and amplifications were performed in 20-μl reaction volumes consisting of 10 μl of SYBR Supermix, 1 μl each of forward and reverse primers (10 μM), 7 μl of nuclease-free water, and 1 μl of cDNA. Cycling conditions were 95°C for 2 min, followed by 40 cycles of 95°C for 15 s and 60°C for 30 s. At the end of the reaction, a melting curve analysis was conducted from 60 to 95°C to ensure the specificity of each primer pair. *BdKr-h1* and *BdMet* relative expression levels were calculated by normalizing their CQ values using α-tubulin (GenBank Accession No: GU269902) and rps3 (XM_011212815) as internal references, as described previously (Shen et al., 2010; Wei et al., 2015). Four biological replicates were performed for each sample collected from each developmental stage and tissue. The experimental data were analyzed with qBase software (Hellemans et al., 2007).

### Methoprene Treatment *in Vivo*

To clarify the effects of methoprene on adult ovary development, 1-day-old adults were selected. Methoprene (Sigma, St. Louis, MO, United States) was dissolved in acetone and diluted to a final concentration of 5 μg/μl (Haq et al., 2010). One microliter (5 μg) of the methoprene solution was dropped onto the pronota of adults, with acetone used as a control. Treated females of different groups were reared in separate cages. For gene expression determination, adults were randomly selected 4, 8, 12, and 24 h after methoprene treatment. Relative expression levels of *BdKr-h1, BdMet, BdVg1*, and *BdVg2* were measured. In addition, 3- to 7-day-old adults were dissected daily, and the corresponding developmental morphology of the ovary (i.e., surface area) was observed and recorded using a Leica M205A stereomicroscope (Leica Microsystems, Wetzlar, Germany).

### RNA Interference (RNAi)

To study the function of *BdKr-h1* and *BdMet* in the oriental fruit fly, RNAi was applied using dsGFP as a control. *BdKr-h1, BdMet*, and *green fluorescent protein* (*GFP*) genes were first amplified using corresponding primers (**Supplementary Table S1**) including a T7 RNA polymerase promoter. The cloned PCR products were used as precursors for dsRNA synthesis using a Transcript Aid T7 High-Yield Transcription kit (Thermo Scientific, Wilmington, DE, United States) in accordance with the manufacturer’s instructions. The resulting dsRNA product was purified using materials in the same kit. dsRNA quality was measured by agarose gel electrophoresis (1.0%), and the concentration was determined using a NanoVue UV–Vis spectrophotometer (GE Healthcare Biosciences). Each dsRNA was dissolved in RNase-free water to a final concentration of 5 μg/μl and stored in a freezer at -70°C. Gene silencing by dsRNA injection was performed on insects housed in a custom-designed cage. Four-day-old female adults were selected and divided into three experimental groups designated as dsGFP, dsMet, and dsKr-h1. Approximately 1.5 μg of dsRNA was injected into each adult abdomen with a Nanoject II Auto-Nanoliter Injector (Drummond Scientific, Broomall, PA, United States). Three biological repeats were performed, and each replicate consisted of 60 female adults. Relative expression levels of *BdKr-h1, BdMet, BdVg1*, and *BdVg2* were then measured at 24 and 72 h after injection. In addition, ovaries of 7-day-old adults were removed after RNAi treatments and positioned in a drop of phosphate-buffered solution on a glass slide. The ovaries were photographed and their phenotypes were recorded as described in the Section “Methoprene Treatment *in Vivo*.” Adult fecundity was systematically observed as well. After RNAi treatment, individual 9-day-old females were paired with a virgin male of the same age at dusk. After successful mating, each male was transferred and fecundity was recorded continuously for 7 days. For the fecundity assay, each group contained four females and six replicates (i.e., 24 females were included for each treatment).

### Rescue Assay by Methoprene Treatment

To explore the effects of methoprene on female adults after RNAi with dsMet/dsKr-h1, a rescue assay was conducted. RNAi-treated adults were divided into two groups: one for RNAi analysis and the other for the rescue assay. Two hours after dsRNA injection, methoprene was applied in accordance with the method described above (section “Methoprene Treatment *in Vivo*”), with acetone serving as a control. Expression levels of JH signaling and *Vg* genes were then measured at 24 and 72 h later. Ovary phenotypes of 7-day-old adults were also observed and recorded using a Leica M205A stereomicroscope (Leica Microsystems, Wetzlar, Germany).

### Statistical Analysis

All experiments included at least three biological replicates. Statistical analyses were performed in SPSS 20.0. One-way ANOVA followed by Tukey’s test was applied to gene expression data to test for significant differences among different developmental stages or tissues. An independent samples *t*-test (*P* < 0.05 and *P* < 0.01) was used to determine the significance of differences between the treatment and control in the dsRNA injection assay. All data are expressed as mean ± standard error (SE).

## Results

### Sequence Analysis and Phylogenetic Tree Construction

**FIGURE 1:**
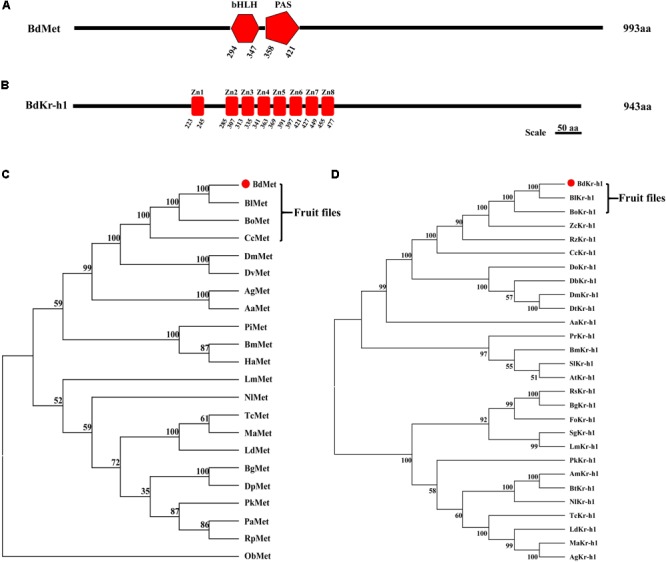
Sequence analysis and a phylogenetic tree of Methoprene-tolerant (Met) and Kr-h1 in *B. dorsalis* and other insects. **(A)** Gene structure of *B. dorsalis* Met, black lines show the length and relative location of characteristic domains. BdMet protein contains bHLH and PAS domains. **(B)** Gene structure of *B. dorsalis* Kr-h1, black lines show the length and relative location of characteristic domain. BdKr-h1 protein contains eight putative zinc-finger domains. The amino acid sequences of BdMet **(C)**, BdKr-h1 **(D)**, and other insect Met and Kr-h1 sequences were selected to analyze the evolutionary relationship using the neighbor-joining method with the MEGA5.0. The numbers at the nodes of the branches represent the level of bootstrap support for each branch. The red dot stands for protein sequence of Met and Kr-h1 from *B. dorsalis.* The Met sequences followed by their GenBank Accession Numbers were listed in the order illustrated: BdMet in *B. dorsalis* (XP_011206298.1); BlMet in *Bactrocera latifrons* (XP_018782574.1); BoMet in *Bactrocera oleae* (XP_014085985.1); CcMet in *Ceratitis capitata* (XP_004527133.1); DmMet in *Drosophila melanogaster* (NP_511126.2); DvMet in *Drosophila virilis* (XP_002055682.1); AgMet in *A. gambiae* (ABC18327.1); AaMet in *A. aegypti* (AAX55681.1); TcMet in *T. castaneum* (NP_001092812.1); MaMet in *Monochamus alternatus* (ANW09588.1); BmMet in *B. mori* (NP_001108458.1); HaMet in *Helicoverpa armigera* (XP_021188262.1); PiMet in *Plodia interpunctella* (ANZ54967.1); LmMet in *L. migratoria* (AHA44478.1); NlMet in *Nilaparvata lugens* (ALT45968.1); PaMet in *P. apterus* (AEW22976.1); BgMet in *B. germanica* (CDO33887.1); PkMet in *Planococcus kraunhiae* (BAU79435.1); DpMet in *Diploptera punctata* (AIM47235.1); PiMet in *Plodia interpunctella* (ANZ54967.1); RpMet in *Rhodnius prolixus* (AEW22977.1); LdMet in *Leptinotarsa decemlineata* (AKG92748.1); ObMet in *Operophtera brumata* (KOB72960.1). The Kr-h1 sequences followed by their GenBank Accession No. were listed in the order illustrated: BdKr-h1 in *B. dorsalis* (XP_011213115.1); BlKr-h1 in *Bactrocera latifrons* (XP_018795146.1); BoKr-h1 in *Bactrocera oleae* (XP_014097247.1); CcKr-h1 in *Ceratitis capitata* (XP_004520990.1); RzKr-h1 in *Rhagoletis zephyria* (XP_017468695.1); ZcKr-h1 in *Zeugodacus cucurbitae* (XP_011193845.1); DmKr-h1 in *Drosophila melanogaster* (NP_477466.1); DtKr-h1 in *Drosophila takahashii* (XP_017006885.1); DoKr-h1 in *Drosophila obscura* (XP_022229324.1); DbKr-h1 in *Drosophila bipectinata* (XP_017097068.1); AaKr-h1 in *A. aegypti* (XP_001655162.2); BmKr-h1 in *B. mori* (NP_001171332.1); SlKr-h1 in *Spodoptera litura* (XP_022820948.1); AtKr-h1 in *Amyelois transitella* (XP_013201221.1); PrKr-h1 in *Pieris rapae* (XP_022122250.1); TcKr-h1 in *T. castaneum* (NP_001129235.1); MaKr-h1 in *Monochamus alternatus* (ANW09587.1); LdKr-h1 in *Leptinotarsa decemlineata* (AGT57869.1); AgKr-h1 in *Anoplophora glabripennis* (XP_018575408.1); NlKr-h1 in *Neodiprion lecontei* (XP_015509259.1); AmKr-h1 in *Apis mellifera* (BAL04728.1); BtKr-h1 in *Bombus terrestris* (NP_001267850.1); BgKr-h1 in *B. germanica* (CCC55948.1); PkKr-h1 in *Planococcus kraunhiae* (BAU79456.1); LmKr-h1 in *L. migratoria* (AHX81747.1); RsKr-h1 in *Reticulitermes speratus* (BAQ21228.1).

Full-length cDNA sequences of *BdMet* (GenBank Accession No. MG763072) and *BdKr-h1* (MG763073) were cloned by RT-PCR from 7-day-old *B. dorsalis* adults. The full-length *BdMet* sequence contained an ORF comprising 2,979 bp encoding a protein of 993 amino acid residues, while the *BdKr-h1* ORF, consisting of 2,829 bp, was predicted to encode 943 amino acid residues. *BdMet* was found to contain bHLH and PAS domains (**Figure 1A**). Among *Met* sequences from analyzed species, bHLH, PAS-A, and PAS-B domains were unique to *TcMet* and *BmMet* (**Supplementary Figure S1**). Of these domains, the PAS domain plays an important role during normal insect functioning. In addition, *BdKr-h1* was found to possess eight putative zinc-finger domains (**Figure 1B**) that were close homologs to those of *Kr-h1* genes of other insect species (**Supplementary Figure S2**). Zn1 and Zn8 were the least conserved of the eight zinc-finger domains of *Kr-h1* (**Supplementary Figure S2**). The predicted product of *BdMet* had a molecular mass of 112.39 kDa and a theoretical isoelectric point of 5.58, while that of *BdKr-h1* had corresponding values of 104.95 kDa and 9.13. No signal peptide was identified in the N- or C-terminal amino acids of *BdMet* or *BdKr-h1*. A phylogenetic tree of *Met* and *Kr-h1* sequences from *B. dorsalis* and other insect species was constructed. According to the phylogenetic analysis, *BdMet* and *BdKr-h1* are very closely related to *Met* and *Kr-h1* from fruit flies of Tephritidae (**Figures 1C,D**).

### Spatiotemporal Expression Patterns

**FIGURE 2:**
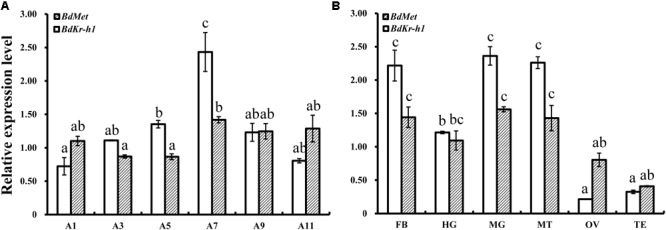
Age- and tissue-specific spatiotemporal expression levels of *BdMet* and *BdKr-h1* in adult *B. dorsalis* individuals. **(A)** Age-specific. A1, A3, A5, A7, A9, and A11 represent 1-, 3-, 5-, 7-, 9-, and 11-day-old adults. **(B)** Tissue-specific. FB, HG, MG, MT, OV, and TE refer to fat body, hindgut, midgut, Malpighian tubules, ovary, and testis of 7-day-old female adults, respectively. Three replicates were conducted, and the data were presented as mean ± SE. Bars with different letters above them differ significantly at *P* < 0.05.

Tissue- and age-specific expression levels of *BdMet* and *BdKr-h1* in adult *B. dorsalis* individuals were determined by qRT-PCR. This analysis revealed that *BdKr-h1* and *BdMet* were both highly expressed in 7-day-old adults, although no significance existed for *BdMet* between 7-day-old and older adults (**Figure 2A**). In addition, *BdKr-h1* and *BdMet* were highly expressed in the fat body, midgut, and Malpighian tubules, and were also expressed in the hindgut (**Figure 2B**).

### Methoprene Treatment

**FIGURE 3:**
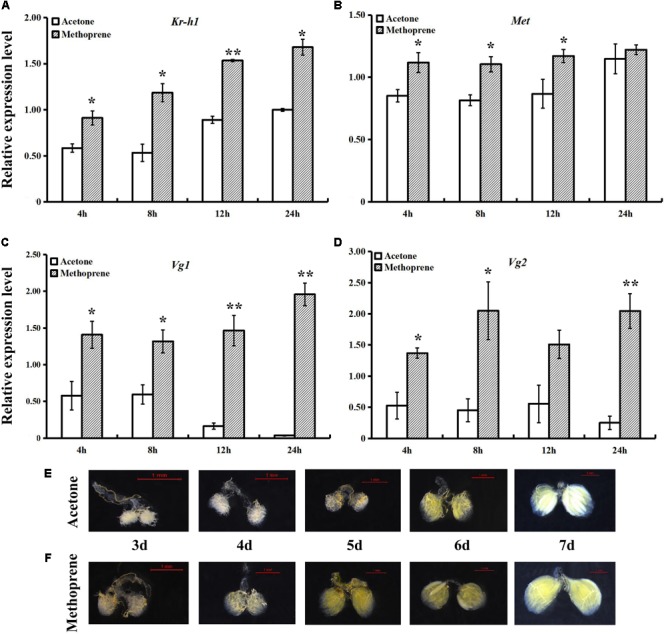
Effects of methoprene on expressions of JH signaling and *Vg* genes and ovary progress. Transcript levels of *BdKr-h1* **(A)**, *BdMet* **(B)**, *BdVg1* **(C)**, and *BdVg2* **(D)** upon methoprene treatment. Three replicates were conducted and the data were presented as mean ± SE. Significant differences between treatment and control were indicated with asterisks (^∗^*P* < 0.05, ^∗∗^*P* < 0.01). **(E)** Ovary progress after acetone treatment and **(F)** methoprene treatment.

After treatment of newly enclosed fruit flies with methoprene, relative expression levels of JH signaling and *Vg* genes (**Figures 3A–D**) were found to be significantly increased relative to those in the controls at almost all time points. The increases in *BdVg1* and *BdVg2* expression after treatment were especially large. Following treatment, ovaries were removed daily from 3- to 7-day-old adults, dissected with special tweezers and photographed. Compared with that in the control group treated with acetone (**Figure 3E**), female ovary development was slightly accelerated by methoprene treatment (**Figure 3F**).

### Involvement of *BdMet* and *BdKr-h1* in Reproduction

**FIGURE 4:**
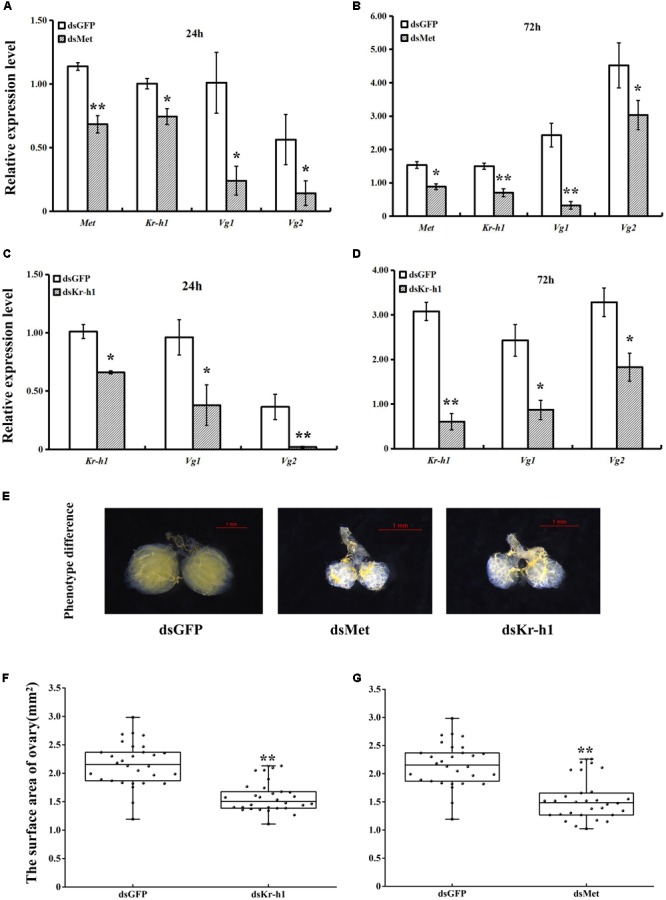
Effects of *BdMet* or *BdKr-h1* silencing on expression levels of JH signaling and *Vg* genes and corresponding ovary observation. Transcript levels of *BdMet, BdKr-h1, BdVg1*, and *BdVg2* 24 h **(A)** and 72 h **(B)** after dsMet injection. Transcript levels of *BdKr-h1, BdVg1*, and *BdVg2* 24 h **(C)** and 72 h **(D)** after dsKr-h1 injection. Three replicates were conducted, and the data were presented as mean ± SE. **(E)** Effects of *Met* and *Kr-h1* RNAi on ovary development with dsGFP as a control. The surface area of ovary recorded after *Kr-h1* RNAi **(F)** and *Met* RNAi **(G)**. Every dot means the surface area of individual ovary. The asterisks indicate statistically significant difference between measurements (^∗^*P* < 0.05, ^∗∗^*P* < 0.01).

**FIGURE 5:**
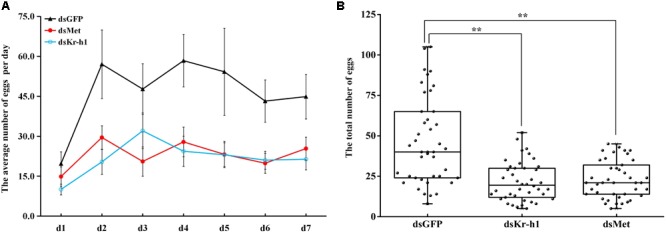
Effects of *Met* and *Kr-h1* RNAi on fecundity in *B. dorsalis*. **(A)** The average number of eggs per day recorded continuously for 7 day after successful mating. Error bars represent the SE of the calculated means based on the six replicates. **(B)** The total number of eggs for 7 days after *Met and Kr-h1* RNAi. Every dot means the total number eggs of four female adults. The asterisks indicate statistically significant difference between treatment and control group (^∗^*P* < 0.05; ^∗∗^*P* < 0.01).

The silencing efficiency of dsMet and dsKr-h1 on JH signaling and *Vg* genes varied significantly over time. The expression of *BdMet, BdKr-h1, BdVg1*, and *BdVg2* was significantly downregulated after injection with dsMet (**Figures 4A,B**). Similarly, silencing of *BdKr-h1* significantly downregulated the expression of *BdVg1* and *BdVg2* (**Figures 4C,D**). Reductions of over 50% of *BdKr-h1, BdVg1*, and *BdVg2* expression occurred 72 h after treatment with dsKr-h1. Subsequent phenotypic observation revealed that silencing of *BdMet* or *BdKr-h1* delayed ovary development (**Figure 4E**), with a consequent decrease in ovary surface area following treatment with dsKr-h1 (**Figure 4F**) or dsMet (**Figure 4G**). Furthermore, analysis of fecundity statistics uncovered reductions in other parameters (i.e., average number of eggs per day and total fecundity) in both dsMet- and dsKr-h1-treated females. Meanwhile, knockdown of *BdMet* or *BdKr-h1* brought about similar phenotype differences (**Figure 5**). These results indicate that JH signaling pathway genes are crucial for reproduction in *B. dorsalis*.

**FIGURE 6:**
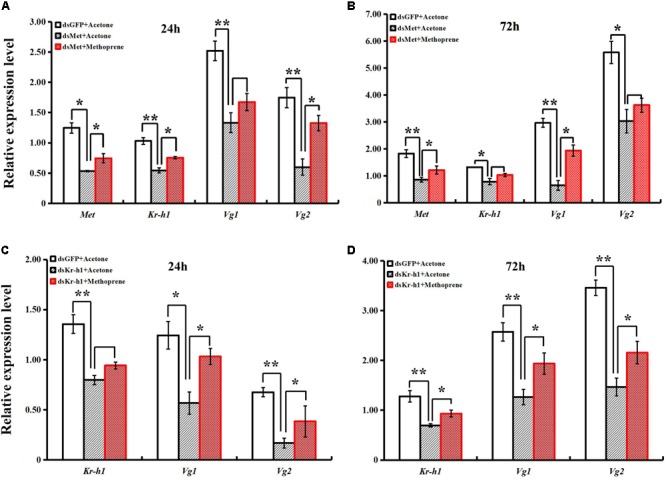
Effects of methoprene treatment on expressions of JH signaling and *Vg* genes from *Met-* and *Kr-h1*-depleted female adults. Methoprene was applied 2 h after dsRNA injection. Transcript levels of *BdMet, BdKr-h1, BdVg1*, and *BdVg2* 24 h **(A)** and 72 h **(B)** after dsMet injection + methoprene with two controls (i.e., dsGFP + Acetone and dsMet + Acetone). Transcript levels of *BdKr-h1, BdVg1*, and *BdVg2* 24 h **(C)** and 72 h **(D)** after dsKr-h1 injection + methoprene with two controls (i.e., dsGFP + Acetone and dsKr-h1 + Acetone). Three replicates were conducted, with the data presented as mean ± SE. Significant differences between treatment and control are indicated with asterisks (^∗^*P* < 0.05; ^∗∗^*P* < 0.01).

**FIGURE 7:**
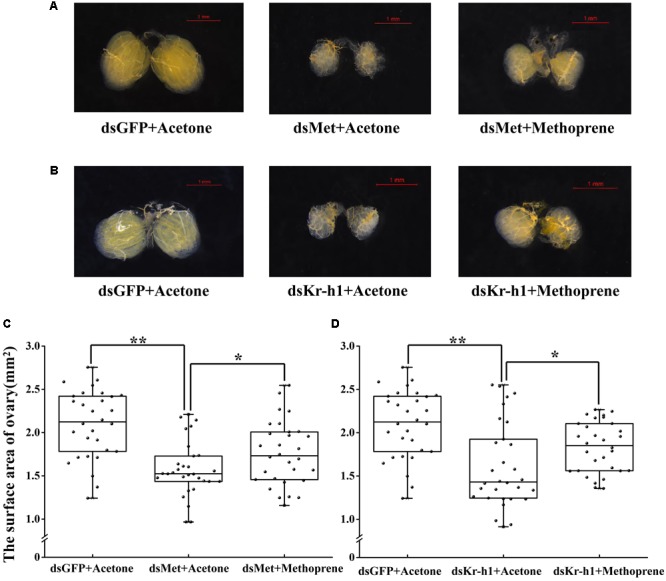
Effects of methoprene treatment on ovary in *Met-* and *Kr-h1-*depleted female adults. Ovary phenotypes of 7-day-old adults were observed and recorded. Methoprene was applied 2 h after dsRNA injection on 4-day-old female adults. Rescue with methoprene after dsMet injection **(A)** with two controls (i.e., dsGFP + Acetone and dsMet + Acetone) and dsKr-h1 injection **(B)** with two controls (i.e., dsGFP + Acetone and dsKr-h1 + Acetone). The surface area of ovary recorded after rescue with methoprene after *Met* RNAi **(C)** and *Kr-h1* RNAi **(D)**. Every dot means the surface area of individual ovary. The asterisks indicate statistically significant difference between measurements (^∗^*P* < 0.05; ^∗∗^*P* < 0.01).

A rescue assay was carried out to further clarify the effects of the JH analog on adult *B. dorsalis* reproduction. The application of methoprene after RNAi significantly stimulated the expression levels of JH signaling pathway and *Vg* genes, and time-dependent effects existed for methoprene application (**Figure 6**). Although there were still phenotypic differences between the ovaries of rescued and control (pre-RNAi) groups, ovary redevelopment with a significantly larger surface area occurred following methoprene rescue after RNAi with *BdMet* or *BdKr-h1* (**Figure 7**). This result is consistent with the methoprene-induced changes in spatiotemporal expression patterns and phenotypes described earlier.

## Discussion

In this study, the full-length cDNA sequences of *Met* and *Kr-h1* were cloned from *B. dorsali*s. Comparison with sequences of other insect species allowed the identification of bHLH and PAS domains in *BdMet* and eight putative zinc-finger domains in *BdKr-h1*. These domains are very similar to those reported in many other insect species. For example, *Met* bHLH and PAS domains have been reported in the red flour beetle, *T. castaneum* (Charles et al., 2011), and in the genus *Drosophila* (Baumann et al., 2010). Recent research has found that *Met* of *T. castaneum* has physiological affinity to JHIII, and the PAS-B domain of *TcMet* is a JH-binding site. These findings are consistent with our results in *B. dorsali*s. The eight putative zinc-finger domains identified in *BdKr-h1* have also been found in other species, such as *A. aegypti* (Shin et al., 2012; Zou et al., 2013), *B. germanica* (Lozano and Belles, 2014), *P. apterus* (Konopova et al., 2011), *Frankliniella occidentalis* (Minakuchi et al., 2011), and *Agrotis ipsilon* (Duportets et al., 2012). Zn1 and Zn8 are the least-conserved zinc-finger domains in *BdKr-h1*, and Zn4 is the shortest. Finally, our phylogenetic analysis demonstrated that *BdMet* and *BdKr-h1* have a close relationship to Tephritidae *Met* and *Kr-h1*.

Juvenile hormone governs many processes in insect development, metamorphosis, and reproduction. At the adult stage, JH promotes reproduction and regulates ovary and accessory gland development (Riddiford, 1994; Wyatt and Davey, 1996). To reveal the molecular basis of JH function, it is thus very important to determine whether *Kr-h1* is involved in JH-stimulated reproduction. In the present study, an examination of spatiotemporal expression patterns of *BdMet* and *BdKr-h1* revealed that these genes were highly expressed in 7-day-old adults, whose ovaries are basically fully developed, with lower expression observed on other days. This result indicates that *Met* and *Kr-h1* of *B. dorsalis* are involved in ovary development and exert different but interacting functions. At the same time, high expression of both *Met* and *Kr-h1* in fat body of mature female adults indicates that the fat body is essential for adult ovary development. This result is consistent with previous findings in locust, in which JH controls the synthesis of Vg in the fat body and its secretion into the hemolymph and uptake by developing oocytes (Song et al., 2014). In *B. germanica*, high expression of *Met* in the fat body implies its involvement in the vitellogenic role of JH (Lozano and Belles, 2014), while transcripts of *Met* and *Kr-h1* are related to oogenetic processes in *A. aegypti* (Zhu et al., 2010). In the brown planthopper *Nilaparvata lugens*, JH receptor Met and its downstream transcription factor Kr-h1 are crucial for ovary development and egg maturation (Lin et al., 2015), and exogenous JH mimic application greatly affects both ovarian development and number of eggs (Chen et al., 2004). In the locust *L. migratoria*, methoprene application of JH-deprived female adults has been observed to stimulate *Kr-h1* transcription approximately fivefold (Song et al., 2014). In our study, treatment with methoprene generally led to the upregulation of JH signaling and the transcription of *Vg* genes. Upregulation of the *Vg* gene is crucial for female ovary development, further implying that *BdKr-h1, BdVg1*, and *BdVg2* are involved in *B. dorsalis* reproduction. Compared with that in the acetone-treated control, ovary development was slightly accelerated after treatment with methoprene in this study.

**FIGURE 8:**
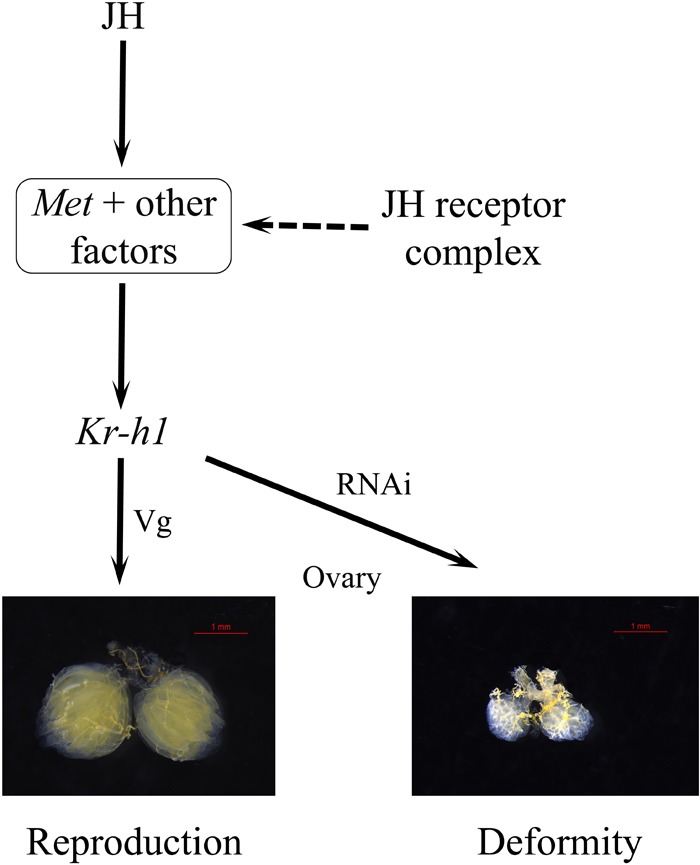
A model for JH signaling in reproduction of *B. dorsalis.* The JH-receptor complex, consisting of Met and other factors, induces the expression of the transcription factor Kr-h1 that subsequently regulate the expression of *Vg* and the reproductive process.

To uncover the roles of *BdKr-h1* and *BdMet* in insect reproduction, we applied RNAi technology to study their physiological functions. *BdMet* and *BdKr-h1* were depleted at the adult stage, which caused female ovary development to be inhibited (**Figure 4**). These experimental results imply that *Met* and *Kr-h1* in *B. dorsalis* are related to the JH signal transduction process that promotes ovary development and egg production. Vitellogenesis and oogenesis in the linden bug, *P. apterus*, are also dependent on JH; however, *Kr-h1* RNAi has no obvious impact on *Vg* expression or ovarian development in the female adults of this species (Smykal et al., 2014), possibly because of incomplete silencing of the *Kr-h1* gene. In contrast, *Met* silencing leads to significant reduction of *Vg* expression and impedes oocyte maturation in *T. castaneum, L. migratoria*, and *P. apterus* (Parthasarathy et al., 2010b; Smykal et al., 2014; Song et al., 2014). In the beetle *T. castaneum*, JH regulates Vg synthesis and ovary growth (Parthasarathy et al., 2010a), and *Kr-h1* RNAi causes an approximately 30% reduction in *Vg* expression in the female adults of this species (Parthasarathy et al., 2010b). *BdVg1* and *BdVg2* expression was significantly downregulated after *Met* and *Kr-h1* RNAi, suggesting that Vg transcription is regulated by *Kr-h1*. These results demonstrate the involvement of *Met* and *Kr-h1* in the JH signaling pathway in *B. dorsalis* to promote ovary development (**Figure 8**), consistent with previously described findings in linden bug (Smykal et al., 2014). In the current study, ovaries in dsKr-h1- and dsMet-treated insects appeared to be developmentally delayed compared with those of the control group. Moreover, the surface area of the ovary and fecundity was significantly lower following RNAi treatment (**Figure 4**). These results provide further evidence that *Kr-h1* is essential for ovary growth and egg production.

To explore the effects of methoprene treatment on *Met-* and *Kr-h1*-depleted female adults, methoprene (dissolved in acetone) was applied to adults after *Met* and *Kr-h1* RNAi. Relative expression levels of JH signaling and *Vg* genes in female adults were differentially upregulated after treatment with methoprene. At the same time, ovary growth in *Met*- and *Kr-h1*-RNAi-treated *B. dorsalis* was partially restored after methoprene treatment, but defective phenotypes were not restored to their normal status (**Figure 7**). However, in some other species like *T. castaneum* and *L. migratoria*, methoprene application after *Met* or *Kr-h1* RNAi can’t rescue a number of phenotypes (Minakuchi et al., 2009; Song et al., 2014). Considering that RNAi, not a transgenic technique, was applied here, it is understandable that methoprene treatment on *Met* RNAi individuals rescued some phenotypes. Although this could have been due to partial reduction of Met by RNAi, this could also suggest that there might be another *Met-like* gene in this species. In *Drosophila* species and in the tsetse fly, both Met and Gce are present (Jindra et al., 2015). Two paralogs of Met/Gce have also been confirmed in *Bombyx mori* (Kayukawa et al., 2012). However, a single Met-like ortholog has been found in *A. aegypti, Culex pipiens*, and *Anopheles gambiae* mosquitoes. Another paralog, Gce, is present in *B. dorsalis.* Owing to the tendency for RNAi technology to achieve partial silencing, the current study involved investigation of the JH receptor *Met*, but not its paralog *Gce*. Among these various molecules, this study focused on the functional analysis of Kr-h1. However, *Gce* in *B. dorsalis* warrants further functional exploration, including in terms of its functional redundancy with Met. Nonetheless, the results of these methoprene experiments indicate that *Met* and *Kr-h1* regulate Vg expression; they further show that *Kr-h1* is important for ovary development and egg production in female adults of *B. dorsalis*. The results of the present study are consistent with the previously described findings of Wyatt and Davey (1996). In addition, the *Kr-h1* expression was induced to approximately threefold higher levels in the vitellogenic phase, which is consistent with the high JH titer during this stage (Dale and Tobe, 1986; Glinka et al., 1995).

In summary, the full ORFs of *BdMet* and *BdKr-h1* from *B. dorsalis* were cloned and phylogenetically analyzed. Spatiotemporal expression analysis of *BdMet* and *BdKr-h1* revealed that both genes were highly expressed in the fat body of 7-day-old female adults. Methoprene treatment significantly upregulated the relative expression of JH signaling and *Vg* genes and slightly accelerated ovary development. The results of our subsequent reverse RNAi experiments imply that JH promotes the expression of *Kr-h1* via *Met* to enhance female ovary development and that *Met* transduces JH signaling to promote ovary growth by maintaining high *Kr-h1* expression. Finally, a rescue assay with methoprene stimulated the expression of JH signaling and *Vg* genes, which partially restored ovary growth, further illustrating the importance of JH in female ovary development. The current data reveal an essential role of *Kr-h1* in insect ovary growth and egg production. Our findings indicate that the JH-Met-Kr-h1 signaling pathway functions in insect reproduction.

## Author Contributions

YY, J-JW, WD, and E-HC designed the research. YY, R-LY, W-PW, and Q-HZ performed the research. All authors analyzed the data and discussed the results during the progress of the work. G-RY, J-JW, and WD contributed the biological samples, reagents, analytical tools, and laboratory equipment. YY and WD wrote the paper with contributions from G-RY and J-JW. All authors gave final approval for the publication.

## Conflict of Interest Statement

The authors declare that the research was conducted in the absence of any commercial or financial relationships that could be construed as a potential conflict of interest.

## Footnotes

1 https://www.ncbi.nlm.nih.gov/

2 http://web.expasy.org/protparam/

3 http://www.cbs.dtu.dk/services/SignalP

## Supplementary Material

FIGURE S1 Comparison of the characteristic domains in BdMet with other insect Met proteins. The protein sequences include BdMet (MG763072), Met from *Drosophila melanogaster* (DmMet: NP_511126.2), *T. castaneum* (TcMet: NP_001092812.1), *A. aegypti* (AaMet: AAX55681.1), and *B. mori* (BmMet: NP_001108458.1). Conservative amino acids were indicated with asterisks, while the bHLH (green areas), PAS-A (red areas), and PAS-B (purple areas) domains of Met were highlighted using black box. In particular, the TcMet and BmMet have two PAS characteristic domains (PAS-A and PAS-B).

FIGURE S2 Comparison of the characteristic domains in BdKr-h1 with other insect Kr-h1 proteins. The protein sequences include BdKr-h1 (MG763073), Kr-h1 from *B. mori* (BmKr-h1: NP_001171332.1), *Drosophila melanogaster* (DmKr-h1: NP_477466.1), *T. castaneum* (TcKr-h1: NP_001129235.1), and *A. aegypti* (AaKr-h1: XP_001655162.2). Conservative amino acids were indicated with asterisks, while the eight putative zinc-finger domains of Kr-h1 were highlighted using black box. They were marked with different colors, respectively.

TABLE S1 Primers used for cDNA cloning, quantitative real-time PCR (qRT-PCR), and double-stranded RNA (dsRNA) synthesis.
