# Supplementary figures and images for: Involvement of Met and Kr-h1 in JH-Mediated Reproduction of Female Bactrocera dorsalis (Hendel)
Source: Front Physiol. 2018 May 4;9:482. doi: 10.3389/fphys.2018.00482 (PMC5945869; doi:10.3389/fphys.2018.00482)

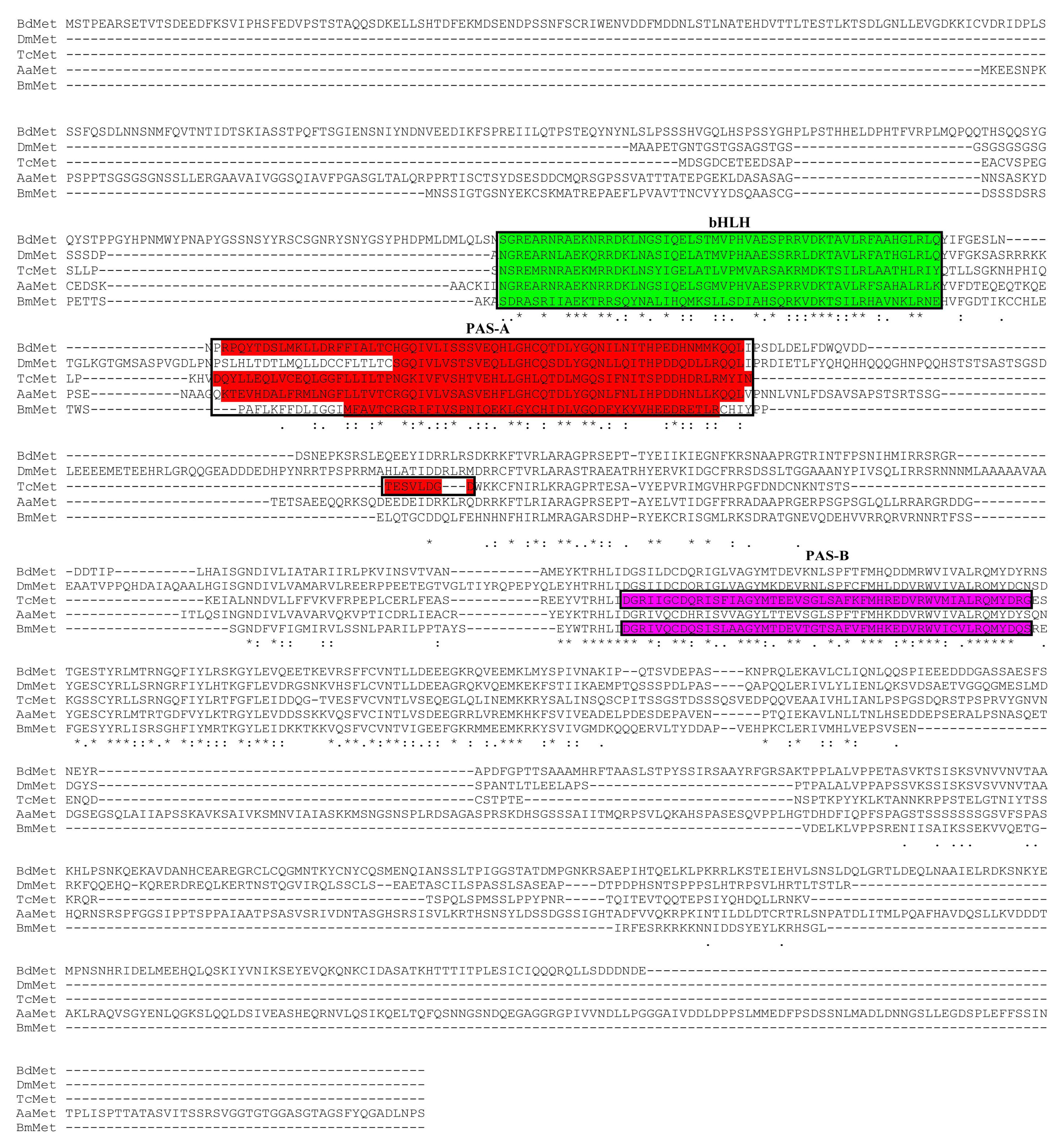

Supplement: FIGURE S1 — Comparison of the characteristic domains in BdMet with other insect Met proteins. The protein sequences include BdMet (MG763072), Met from Drosophila melanogaster (DmMet: NP_511126.2), T. castaneum (TcMet: NP_001092812.1), A. aegypti (AaMet: AAX55681.1), and B. mori (BmMet: NP_001108458.1). Conservative amino acids were indicated with asterisks, while the bHLH (green areas), PAS-A (red areas), and PAS-B (purple areas) domains of Met were highlighted using black box. In particular, the TcMet and BmMet have two PAS characteristic domains (PAS-A and PAS-B). [file Image_1.JPEG]

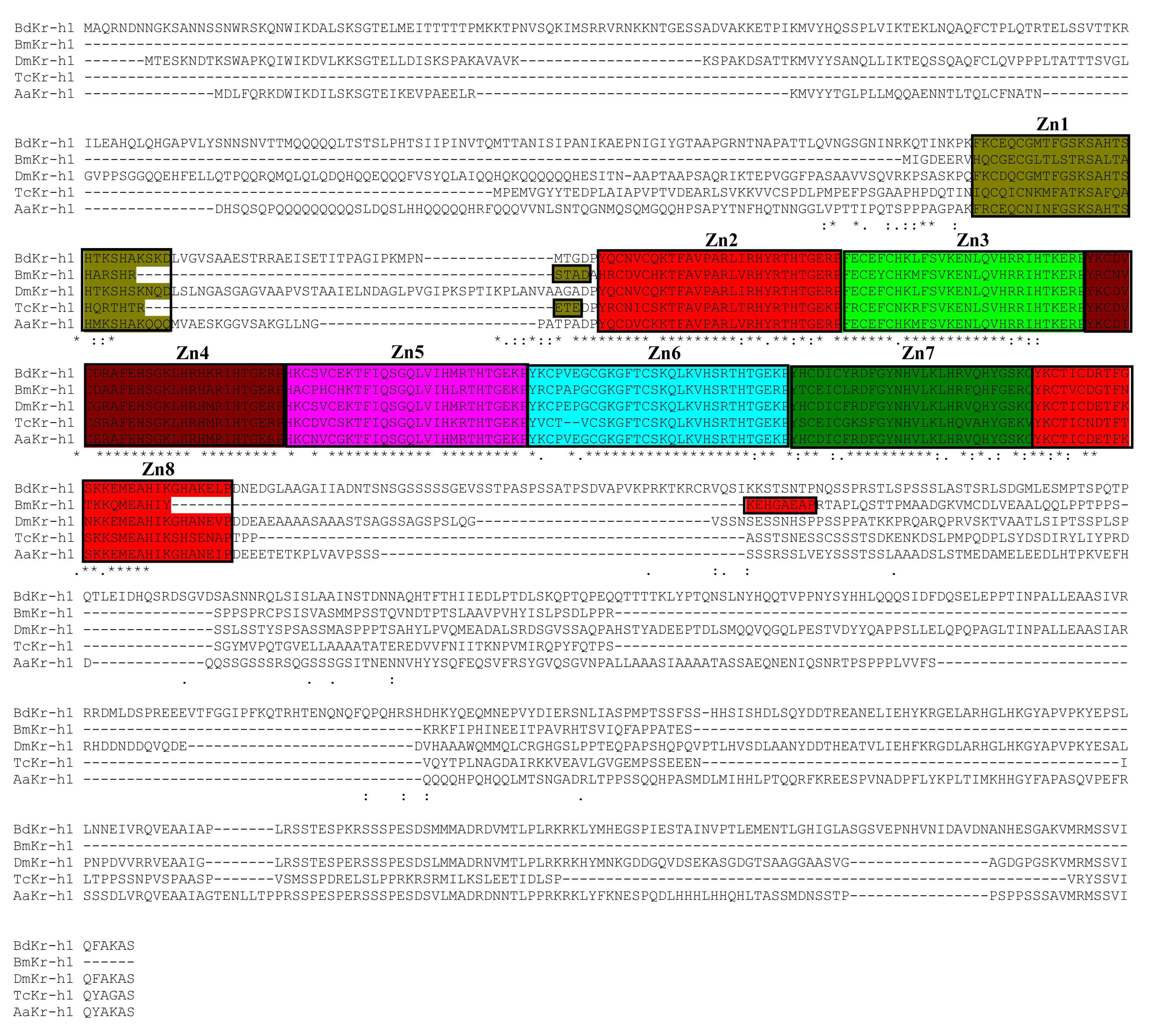

Supplement: FIGURE S2 — Comparison of the characteristic domains in BdKr-h1 with other insect Kr-h1 proteins. The protein sequences include BdKr-h1 (MG763073), Kr-h1 from B. mori (BmKr-h1: NP_001171332.1), Drosophila melanogaster (DmKr-h1: NP_477466.1), T. castaneum (TcKr-h1: NP_001129235.1), and A. aegypti (AaKr-h1: XP_001655162.2). Conservative amino acids were indicated with asterisks, while the eight putative zinc-finger domains of Kr-h1 were highlighted using black box. They were marked with different colors, respectively. [file Image_2.JPEG]
