# Supplementary material for: Involvement of Met and Kr-h1 in JH-Mediated Reproduction of Female Bactrocera dorsalis (Hendel)
Source: Front Physiol. 2018 May 4;9:482. doi: 10.3389/fphys.2018.00482 (PMC5945869; doi:10.3389/fphys.2018.00482)
Supplement: TABLE S1 — Primers used for cDNA cloning, quantitative real-time PCR (qRT-PCR), and double-stranded RNA (dsRNA) synthesis. [file Table_1.DOC]

**Table S1. Primers used for cDNA cloning, quantitative real-time PCR (qRT-PCR) and double-stranded RNA (dsRNA) synthesis.**

| **Target** | **Direction** | | **Nucleotide Sequence 5’ to 3’** |
| --- | --- | --- | --- |
| *BdMet* (Cloning) | Forward | ATGAGCACACCTGAAGCACG | |
| *BdKr-h1* (Cloning)  M13(Cloning) | Reverse  Forward  Reverse  Forward  Reverse | CGGACTTAATCGAGATTATTCATC  ATAATGGCACAACGCAAC  CAAGTGACTTCAAATGAT  GCCAGGGTTTTCCCAGTCACGA  GAGCGGATAACAATTTCACACAGG | |
| *BdMet* (qRT-PCR) | Forward | GGAACTGCTTAGCCATACCG | |
|  | Reverse | GCCCAAGTCCGATGTTTTTA | |
| *BdKr-h1* (qRT-PCR) | Forward | TGCCAACATCAAAGCAGAAC | |
|  | Reverse | CGCGGAAACACCAACTAAAT | |
| *BdVg1* (qRT-PCR) | Forward | CAACCAATCAGCAATAACCAGGAC | |
|  | Reverse | GCATCACCACGAGCCAAACC | |
| *BdVg2* (qRT-PCR) | Forward | CAGGAAGGAGAGCGTTTGATTGG | |
|  | Reverse | CTGTTGTCCGTAGTAGCGTTGC | |
| *BdMet* (dsRNA) | Forward | taatacgactcactataggg TGACCCCAGCTCTAACTTCAG | |
|  | Reverse | taatacgactcactataggg ACGGCGAATGCAATTGACCA | |
| *BdKr-h1*(dsRNA) | Forward | taatacgactcactatagggAAATGGCAGCGGCAACATAAA | |
|  | Reverse | taatacgactcactataggg AAAGCTCGGTCGCAGACAT | |
| *dsGFP* (dsRNA) | Forward | taatacgactcactatagggCAGTTCTTGTTGAATTAGATG | |
|  | Reverse | taatacgactcactatagggTTTGGTTTGTCTCCCATGATG | |
| *α*-*Tubulin* (internal reference) | Forward | CGCATTCATGGTTGATAACG | |
| rps3 (internal reference) | Reverse  Forward  Reverse | GGGCACCAAGTTAGTCTGGA  TGGATCACCAGAGTGGATCA  TAAGTTGACCGGAGGTTTGG | |
